# Towards Monitoring and Identification of Red Palm Weevil Gender Using Microwave CSRR-Loaded TL Sensors

**DOI:** 10.3390/s23156798

**Published:** 2023-07-30

**Authors:** Mohammed M. Bait-Suwailam

**Affiliations:** 1 Remote Sensing and GIS Research Center, Sultan Qaboos University, Muscat P.C. 123, Oman; msuwailem@squ.edu.om; 2 Department of Electrical and Computer Engineering, Sultan Qaboos University, P.O. Box 33, Muscat P.C. 123, Oman

**Keywords:** date palm, CSRR, metamaterials, microwave sensor, red palm weevil

## Abstract

This paper presents for the first time the design of a microwave sensing setup for the potential monitoring and identification of red palm weevil (RPW) gender type. The microwave sensor consists of a planar two-port transmission line (TL) with a single complementary split-ring resonant (CSRR) inclusion etched from the bottom metallic layer. The CSRR sensor is placed on top of a customized non-conductive container. The microwave sensing setup was designed, numerically demonstrated, fabricated and tested experimentally. Simulated results correlate quite well with the experimental data. Moreover, the sensitivity of the CSRR sensor when in close proximity to different RPW genders was evaluated both numerically and experimentally. Based on the measured results from 15 RPW samples with different body sizes, different RPW gender types showed unique microwave signatures. A notable shift in the sensor’s resonance frequency was achieved, where on average a resonant frequency shift of 10% for adult RPWs was achieved, while a 2.4% frequency change was obtained for larvae (young) RPWs. Hence, the proposed microwave sensing setup can be adopted in field trials to examine and differentiate between various RPW genders at various developmental stages.

## 1. Introduction

Among many harmful insects, red palm weevil (RPW), also known as Asian palm weevil, is considered a big threat to date palm trees, especially in the Middle East and North Africa (MENA) region. Such pests have the ability to rapidly grow in a short period of time, adopt themselves easily inside the palm trunk in various environmental conditions and can move (travel) from one place to another over several kilometers due to their ability to fly. Unfortunately, the rapid spread of RPW pests results in severe damage to date palm trees, which in turn causes major losses to the economic growth of many developing countries, including many countries within the MENA and Asian regions.

**Figure 1:**
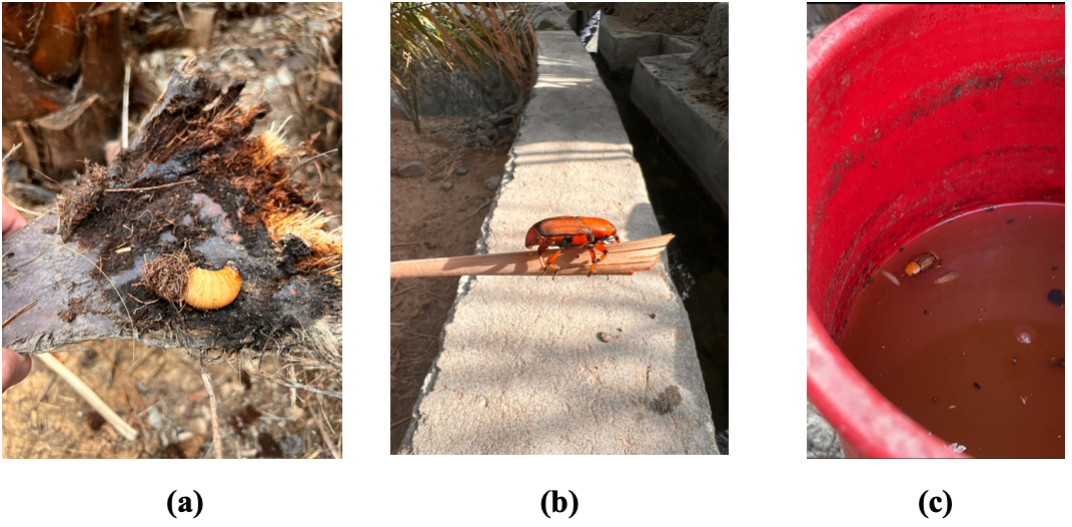
Photos taken from a local date palm farm, showing collected palm insects from infested palm tree (**a**) larva, (**b**) adult RPW and (**c**) an adult RPW captured inside of a prepared field trap.

One commonly adopted management control technique for RPW pests is the use of traps. The traps are prepared by filling a container with an insect’s pheromone mixed with palm odor and water [1,2]. The traps can easily attract RPWs due to the strong effect of both substances (pheromone mixture with palm odor) in attracting other RPWs. Figure 1a,b depict photos of two collected palm weevil larva and an adult RPW, while Figure 1c shows a captured RPW inset inside a prepared trap in a red container in a local farm.

There have been numerous research studies and efforts in the literature devoted to managing and controlling the spread of red palm weevil pests, including detecting the presence of RPW pests in palm trunks using acoustic signal processing [3], optical distributed sensing [4,5], high power microwave sensing and thermal heating treatment procedures [6,7,8,9,10], among other techniques. The research work in [4] presented a technique for monitoring and detecting RPWs by using optical-fiber-distributed acoustic sensing probes. The sensing system in [4] was able to detect the feeding activities of almost 12-day-old RPWs: the larvae in an infested palm tree. Massa et al. developed in [6] a high-power microwave-based heating system as a potential modality to control and treat palm trees for the invasive RPW. The study in [6] also presented a numerical thermal study in order to estimate the amount of time required to completely destroy red palm weevil pests. Among the numerical simulation developments related to the control of RPWs is the work presented in [8], where authors presented an FDTD simulation model to study the effectiveness of microwave energy in isolated RPW irradiation. Based on the numerical simulation results, RPWs at larvae stage were found to absorb sufficient electromagnetic energy that led to the complete destruction of RPWs. Another numerical study of the microwave heating of RPWs was presented in [9], in which the authors developed an array of Vivaldi antenna. In the study, the main focus was on the thermal treatment of the infested palm trees through the use of high-power microwaves. Based on the findings from [6,9], it was concluded that high-power microwaves are an effective mechanism for the reliable destruction of palm weevils.

To the best of our knowledge, there have been no major developments or comprehensive studies for the identification of RPW gender type, especially with the help of low-power microwave sensors. The contributions in this research study are summarized below:

1. Develop microwave CSRR-loaded transmission line (TL) sensors for the potential identification and detection of RPW gender type through the recording of the transmission strength of the sensor.
2. Present 3D numerical models that mimic the nature of microwave CSRR sensors in the detection of various RPW genders.
3. Present an experimental setup of the microwave identification setup for RPW gender detection. Due to the limited access to a collection of RPWs, only samples were considered in the experimental study.
4. Earlier studies focused on the microwave heating treatment of date palm trunks infested with RPW pests. However, this research work explores deeply the effectiveness of the microwave CSRR probes when interacting with RPW pests towards the identification of different gender types of RPWs.
5. This study also assesses the performance of the microwave CSRR sensor over a population of 17 RPW samples that were collected from a local infected date palm farm: 5 samples for adult male RPW, another 5 for adult female RPWs and 7 samples for larvae insects.
6. This study focuses mainly on isolated RPW samples of various gender types, which is important to quantify the strength and effectiveness of the developed microwave sensor. Future studies will aim to investigate the applicability of machine learning on estimating RPW gender type in a more populated area with different RPW genders and species at different stages. Furthermore, this research study contributed to the field of RPW pest control through the deployment of low-power microwaves to investigate the potential identification of RPW gender instead of the high-power microwave treatments, which were reported in earlier studies; thus, avoiding the need to use harmful radiation that farmers could be exposed to during their daily work.

Due to the vast variations in the electric properties of the RPW at its different life development stages, we believe that microwave CSRR transmission-line-based sensors are attractive to deploy. This is due to their low profile, ease of fabrication and narrowband sensing capability. The microwave sensor is placed on top of the sensed RPW pest with a small controllable stand-off distance. In this study, numerical full-wave simulations are presented and validated with experimental results, where all the materials’ properties of the setup including the RPW types are included in the simulation models.

## 2. Numerical Simulations of RPW Gender Identification

### 2.1. Numerical Modeling of the CSRR Sensor

**Figure 2:**
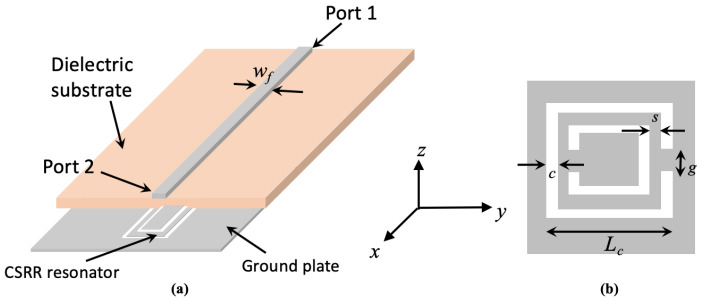
(**a**) Schematic of the CSRR probe, (**b**) the complementary split-ring resonator with its design parameters. The grey area represents metallization.

In the past twenty years, the integration of small-resonant metamaterial particles, in the form of split-ring resonators (SRRs) [11] or complementary split-ring resonators (CSRRs) [12,13], in planar microwave transmission lines has led to numerous developments of and enhancements to sensory elements and devices in many applications, including but not limited to radio frequency microwave circuits, and materials’ characterization and classification [14,15,16,17]. Complementary SRR can be regarded as a quasi-static resonator when its dimension is electrically small as compared with the operating wavelength of the excitation electromagnetic field. In such a case, the microwave CSRR resonance frequency is attributed to the equivalent LC-resonant tank circuit upon an excitation of an external normal electric field component to the resonator’s surface area, i.e., *z*-direction in Figure 2, where the current flow circulating around the CSRR metallic rings contributes to the inductance *L*, while the cuts (slots) in the two rings result in a developed distributed capacitance effect *C*. The resonance frequency of the CSRR can then be estimated using

(1) $$f_{res}=\frac{1}{\left(2\pi \right)\sqrt{L_{dist}*C_{dist}}}$$

where $f_{res}$ is the resonant frequency of the CSRR sensor, $L_{dist}$ and $C_{dist}$ are the total distributed inductance and capacitance of the CSRR sensor, respectively. The dimensions of the designed microwave CSRR unit inclusion (see Figure 2b) were initially estimated using the relations provided in [18,19,20] based on the resonance frequency of the CSRR from the retrieved effective electric permittivity response. A slight shift in the resonance frequency would be expected since the CSRR resonator is coupled to the transmission line. As such, optimized dimensions are then obtained through trained simulation tasks, which requires the consideration of the capacitive coupling between the rings for a more accurate estimation of the sensor’s resonance frequency.

**Figure 3:**
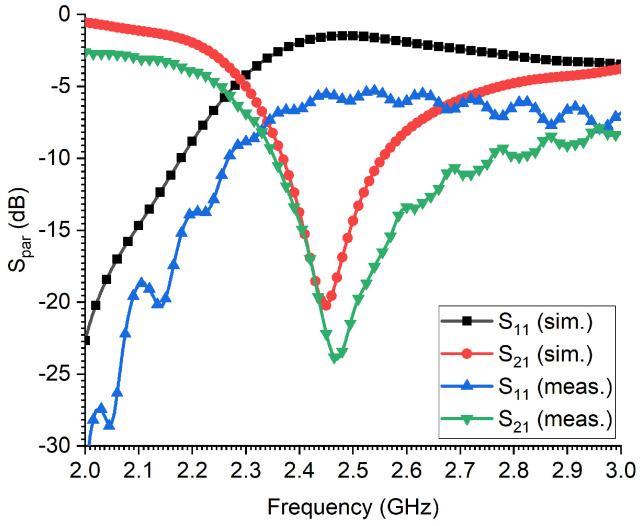
The simulated reflection and transmission coefficients, $S_{11}$ and $S_{21}$, respectively, of the developed transmission-line-based CSRR sensor.

In this work, we deploy a microwave CSRR transmission-line-based sensor as a promising near-field probe for identifying the gender of an RPW, taking into account the advantages of such sensors in terms of their high sensitivity and narrowband operational band. Figure 2a shows the microstrip transmission line CSRR sensor, where a single CSRR unit cell (comprising of double concentric square rings with cuts in opposite sides) is etched out from the metallic solid ground layer, with a transmission line width of $w_{f}$ = 3.05 mm corresponding to a 50 Ω port impedance. Figure 2b shows a schematic for a complementary split-ring resonator unit cell. The optimized dimensions of the CSRR sensor are: $L_{c}$ = 9 mm, *s* = 1.575 mm, *c* = 0.45 mm and *g* = 0.5 mm, where its resonance frequency is 2.45 GHz, within the ISM frequency band. For convenience, an FR-4 square laminate of dimension 6 × 6 mm^2^ (with a dielectric constant of 4.4 and a loss tangent of 0.02) and thickness of 1.6 mm was chosen as the host medium of the microwave CSRR sensor. Figure 3 shows the scattering parameters’ response to both the reflection and transmission coefficients of the modeled microwave CSRR-TL sensor. The sensor resonates quite well at the 2.45 GHz-ISM band, as can be seen from the $S_{21}$ response, with a dip of −20 dB. Good agreement of the sensor’s performance can be seen between the simulated and measured results, despite a slight shift in the measured resonance frequency of less than 15 MHz. The slight discrepancy between the simulated and measured results is attributed to the cable losses.

### 2.2. Numerical Setup of the RPW Gender Detection System

**Figure 4:**
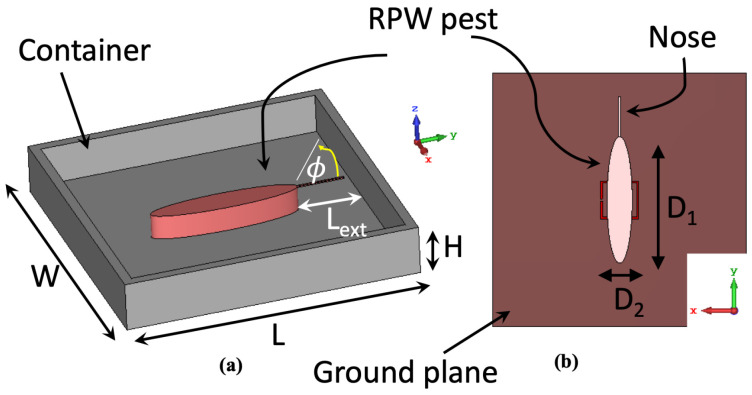
The developed numerical setup to study variations in detecting RPW pest gender type: (**a**) perspective view of the container with the modeled adult RPW, (**b**) bottom view of the microwave CSRR sensor with the RPW pest.

Figure 4 depicts the numerical setup showing a 2 mm thick rectangular container, (mimicking a small cardboard, with a dielectric constant of 2.7). For convenience, the dimensions of the container used are: *L* × *W* × *H* = 60 × 60 × 10 mm^3^. An adult RPW is modeled here as an elliptical cylinder ($D_{1}$ × $D_{2}$ × 1.5 mm) and is placed at the center of the container from its base. In order to have a more realistic scenario for the modeled adult RPW, a thin nose was attached to the body of the pest with a length of $L_{ext}$ = 10 mm.

**Figure 5:**
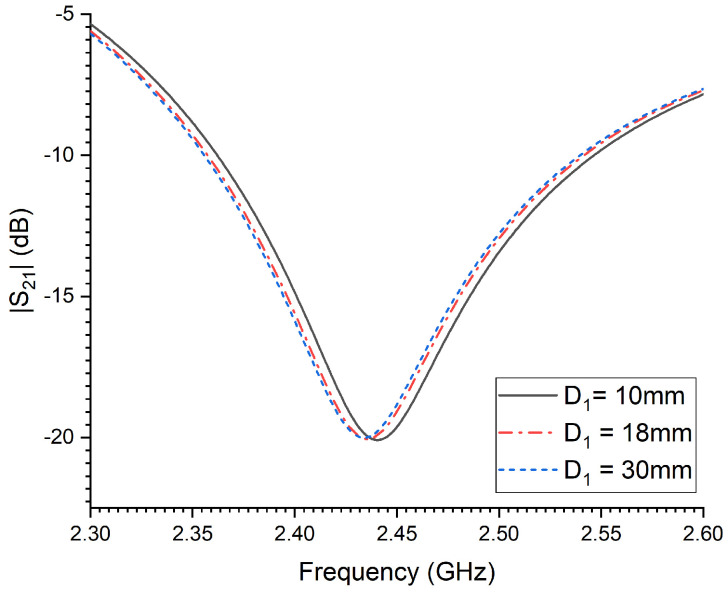
The percentage shift in the sensor’s resonance as a function of the adult RPW pest length, $D_{1}$.

Next, we present a numerical parametric study to investigate the strength of the developed CSRR sensor in predicting changes in the RPW’s size, where $D_{2}$ is kept constant at 6 mm, while $D_{1}$ is arbitrarily varied. The incurred percentage shift in the sensor’s resonance frequency can be estimated using

(2) $$F_{res}Change=\frac{\left(F_{ref}-F_{RPW}\right)}{F_{ref}}\times 100\%$$

where $F_{res}Change$ is the percentage shift in the resonant frequency of the probe in % when the sensor is in close contact with the RPW insect, $F_{ref}$ is the resonance frequency of the probe alone (unloaded), while $F_{RPW}$ is the resonance frequency of the probe when in contact with the RPW. Figure 5 depicts the percentage shift of the sensor’s resonance frequency when in close proximity to the modeled adult RPW when the size of the pest $D_{1}$ is increased from 6 mm to 30 mm. Clearly, there is an increase in the sensitivity strength of the probe as the aperture size of the RPW is increased, which is quantified through recording the sudden horizontal resonant frequency shift of the transmission coefficient minimum (dip).

**Figure 6:**
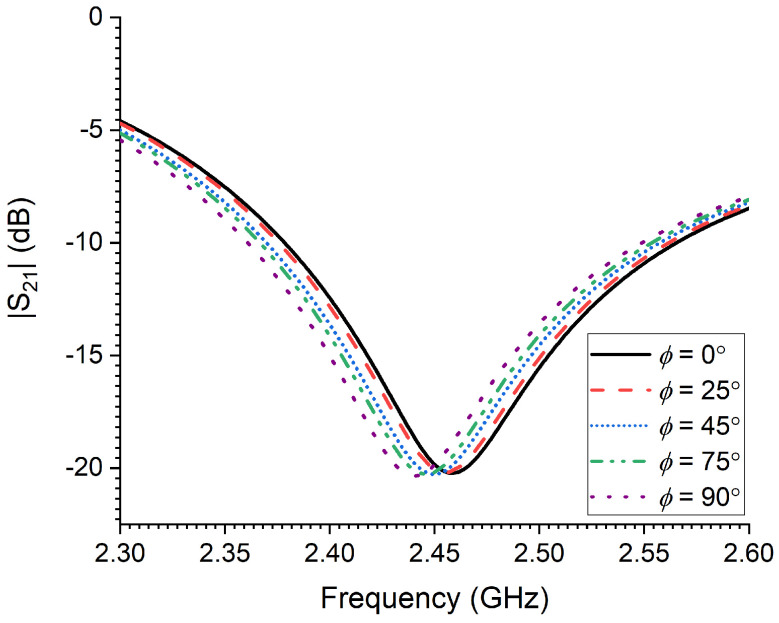
The simulated transmission coefficient of the sensor as a function of RPW larva orientation (rotational movement, ϕ).

It is very important to consider all dielectric losses associated with the modeled RPW at their development stages, namely: larva, pupal chamber and adult. The dielectric constant and loss factor of three RPW genders were estimated from earlier measured data in [21] at 2.45 GHz, which are summarized and presented in Table 1. Figure 6 presents the numerical results of detecting movements of a small RPW larva, where a horizontal rotational movement, i.e., in terms of angle ϕ in Figure 4a, is varied within the xy-plane. It can be seen from Figure 6 that the CSRR resonance frequency tends to shift to lower frequencies as the rotational angle ϕ is increased from 0° to 90°, where the angle of 90° corresponds to an orientation that is perpendicular to the transmission line segment of the sensor. Moreover, the tendency of the sensor’s detection is quite noticeable and appreciable. Hence, the findings from this numerical study are valuable in tracking small movement activities of the RPW, especially during feeding stages.

**Figure 7:**
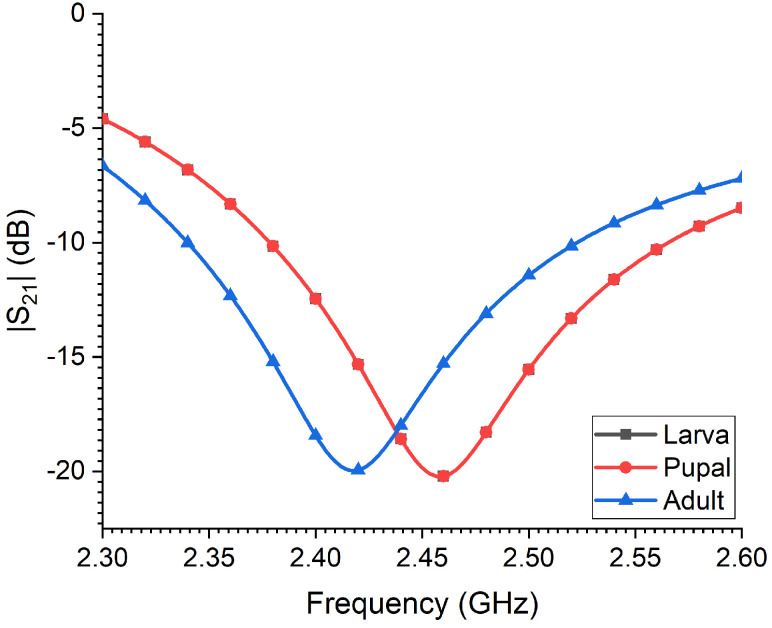
The simulated transmission coefficient of the sensor for modeled RPW at three different development stages: larva, pupal chamber and adult.

Figure 7 depicts a numerical study showing the sensing capability of the CSRR probe in identifying variations in an RPW at its various development stages. A 40 MHz shift in the sensor’s resonance frequency is achieved, which is its ability to differentiate between an adult RPW and either larva or pupal chamber. This is attributed to the reduced water content and moisture of the adult RPW as compared to the larva and chamber. Moreover, from the simulated results, there was no noticeable change in the sensor’s resonance frequency for the cases of the larva and pupal chamber due to the similarities in the body shape of both young insects and a similarity in their electric properties.

**Figure 8:**
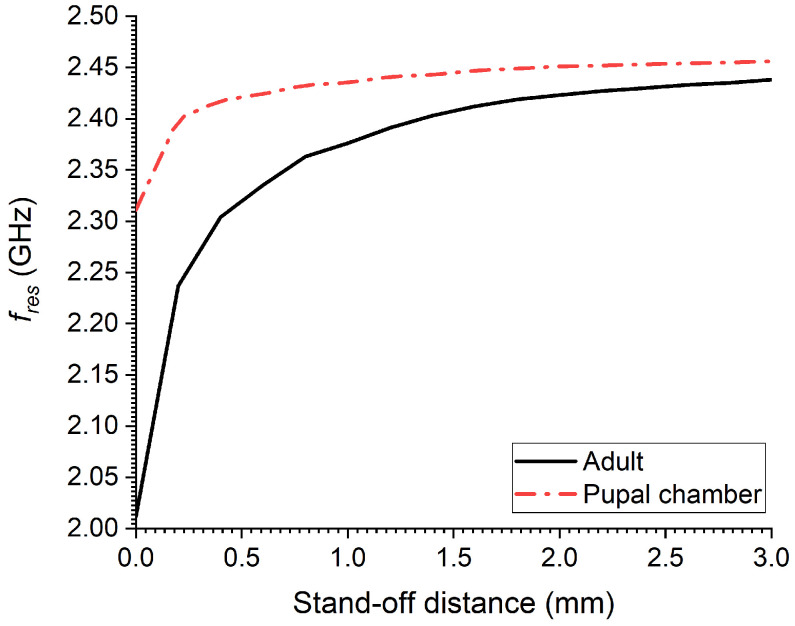
The simulated transmission coefficient of the sensor for modeled RPW at three different development stages: larva, pupal chamber and adult.

Figure 8 presents the simulation results from a numerical parametric study showing the effect of the stand-off distance, i.e., vertical distance between the top (body) surface of the RPW insect and the CSRR ground layer (bottom layer of sensor). In this parametric study, an adult and a pupal chamber were considered. As can be seen, the shift in CSRR resonance frequency is quite significant for the adult case as compared to the pupal case when the sensor is touching both the adult and the pupal insects, stand−offdistance=0.0. The sudden resonance shift in the case of the adult RPW is due to the minimal inherent dielectric losses of the adult RPW as compared with a young insect. Furthermore, both cases reach minimal shift in resonance as the vertical stand-off distance is increased, which was kept at 3 mm for convenience. As such, a stand-off distance between 0.5 mm and 1.5 mm could be used in order to observe higher sensitivity, especially for the case of young RPW insects since they tend to absorb a significant amount of microwave energy and hence reduce their sensitivity strength.

## 3. Experimental Results

**Figure 9:**
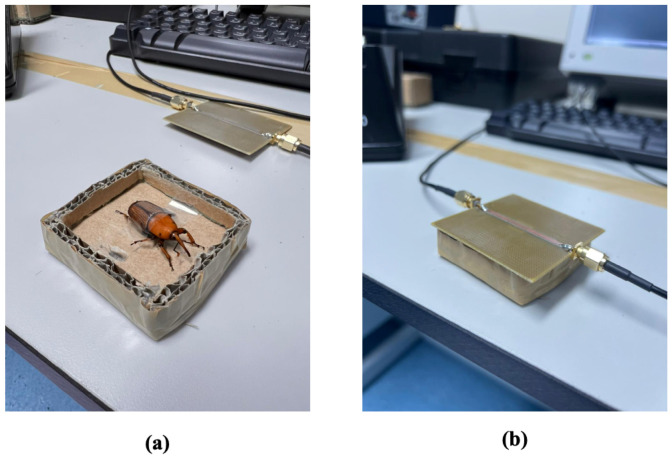
Two photos demonstrating the experimental setup, (**a**) male RPW kept in place inside a paper-based container, (**b**) the microwave CSRR sensor on top of the male RPW pest.

Figure 9 depicts the experimental setup of the RPW gender identification using the developed CSRR sensor. In Figure 9a, an adult RPW (collected from an infested palm tree) was attached to the base of a thin paper container using a thin non-conducting tape. The microwave CSRR sensor was fabricated in-house using a CNC milling machine with two edge-SMA connectors attached to the 50 Ω transmission line for measurement. After calibration, the fabricated sensor was then placed on top of the container and the two ports were connected to the vector network analyzer in order to measure its transmission coefficient.

**Figure 10:**
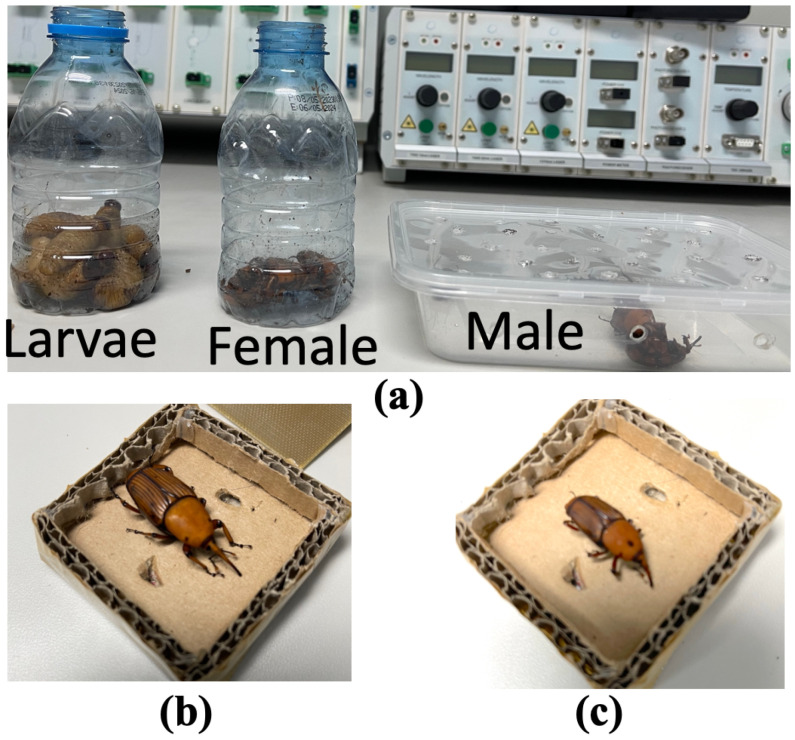
(**a**) The collected RPW samples considered in this study (male, female and larvae RPWs), (**b**) a big size adult male RPW, and (**c**) a small size adult male RPW.

In order to ensure reliability of the microwave CSRR sensor’s sensing capability, a population of 17 RPW pest samples were collected from a local date palm farm, where 10 samples for adult RPWs were collected (5 for each male and female RPWs) and 7 samples from larva pests were collected in maintained air flow containers, as can be seen from Figure 10. Note that it is possible to differentiate between male and female adult RPWs while collecting the samples in the field by observing the little brownish hair in the nose of the male RPW unlike the female RPW, which does not have such hair present in its snout. A magnifier was used for that purpose. Due to the rapid movement and activity of the RPW pests under testing, it was necessary to use a light flexible transparent tape in order to gently maintain the RPW under test and keep it within a referenced location inside the sensing container as illustrated in Figure 9. Table 2 presents the summary of the CSRR sensor’s percentage frequency shift using Equation (2) from the experimental results conducted on 15 RPW samples (5 samples for each of male, female and larvae). The averaged percentage change was around 10% for adult RPWs (male/female), while it was around 2.4% for young larvae insects.

For calibration purposes, the performance of the sensor was first recorded alone, after which, the sensor was assessed when exposed to some selected RPW samples in order to sense both the back and front body surface. Table 3 presents the resonant frequency of the CSRR sensor. As can be seen from Table 3, there were no major changes in the sensor’s resonance frequency from either the front (wings side) or back (belly side) surfaces of the RPW samples, except for there being less than 60 MHz frequency between the front and back sides of the three tested samples.

**Figure 11:**
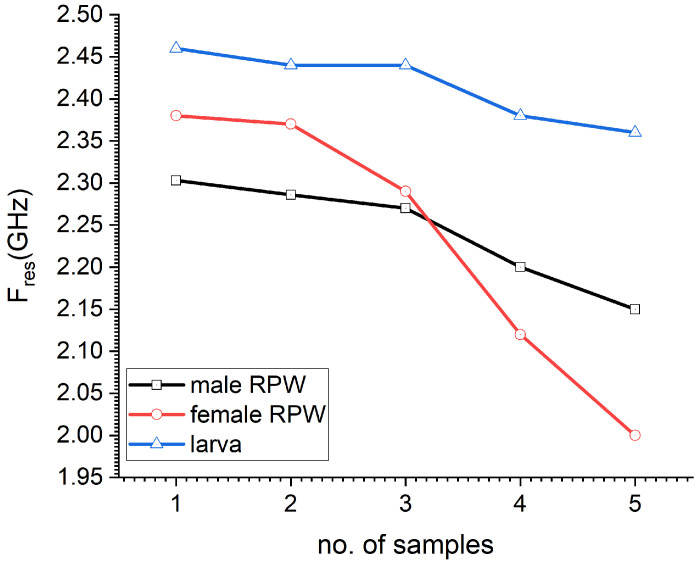
The measured resonance frequency of the sensor for 15 samples of RPW gender, with 5 samples for each male, female and larva pests.

Figure 11 depicts the measured sensor’s resonance frequency per sample under test, where sample numbers are given in order as per the insect’s size and body shape. In other words, sample 1 corresponds to an RPW insect with the smallest possible size within the samples’ collection (whether male, female or larva),while sample 5 corresponds to the biggest RPW under test from the samples collection. For comparison purposes, five samples from larva were also selected. As can be seen from Figure 11, the sensor’s resonance frequency shows minimal changes in the case of sensing the whole five samples of larva, which is expected since larvae do have a cylindrical body with minimal changes in their body size, as compared to the adult RPW (male or female). Furthermore, there was a big shift in the sensor’s resonance frequency in the case of female RPW sample 5, which corresponded to quite a large sized insect.

**Figure 12:**
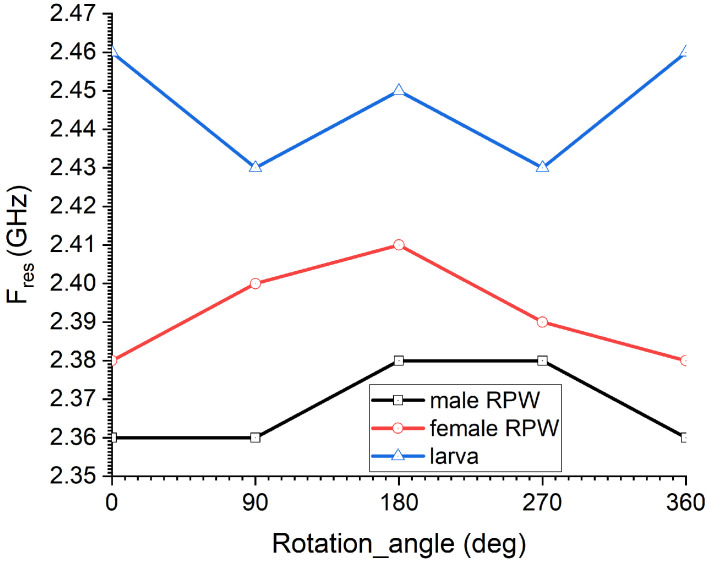
The measured resonance frequency of the sensor as a function of the rotational angle, ϕ, where angle ϕ is illustrated in Figure 4.

Figure 12 presents the measured resonance frequency of the CSRR sensor when exposed to a single RPW sample under four potential rotational mechanisms (see Figure 4): 0°, 90°, 180°, 270° and 360°, which reflects the movement of the RPW insects. From the presented measured data, we can see minimal changes in the detected resonance of the CSRR sensor, which was less than 70 MHz. This is justified due to the variation in electromagnetic field distribution when exposed to the RPW sample under the rotational movement cases.

**Figure 13:**
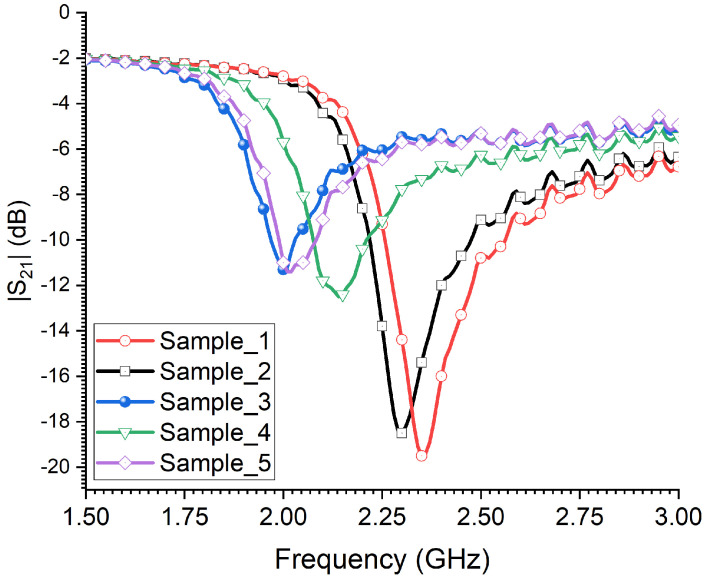
The measured transmission coefficient of the sensor when exposed to 5 adult male RPW insects with different body sizes.

Figure 13 depicts the measured transmission coefficient of the microwave CSRR sensor as a function of frequency for five samples of adult male RPWs. We can see that the sensor’s resonances for samples 4 and 5 have not changed since the two last samples of male RPWs are comparable in size. Moreover, a similar trend in the sensor’s detection strength was observed for the five female RPW samples under testing, in which an increased frequency shift to the resonant frequency towards a lower frequency regime was seen as the female RPW’s size increased.

**Figure 14:**
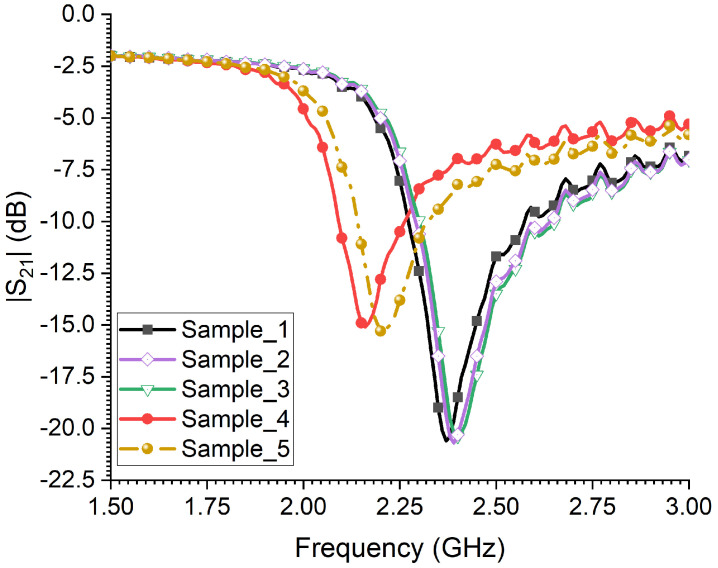
The measured transmission coefficient of the sensor when exposed to 5 adult female RPW insects with different body sizes.

The assessment of detecting adult female RPWs’ gender using the CSRR sensor can be seen in Figure 14. In this case, the sensor has not recorded many changes in its resonance frequency for the first three samples when compared with the last two samples, i.e., samples 4 and 5. This is because the first three samples were quite comparable in body shape and size.

**Figure 15:**
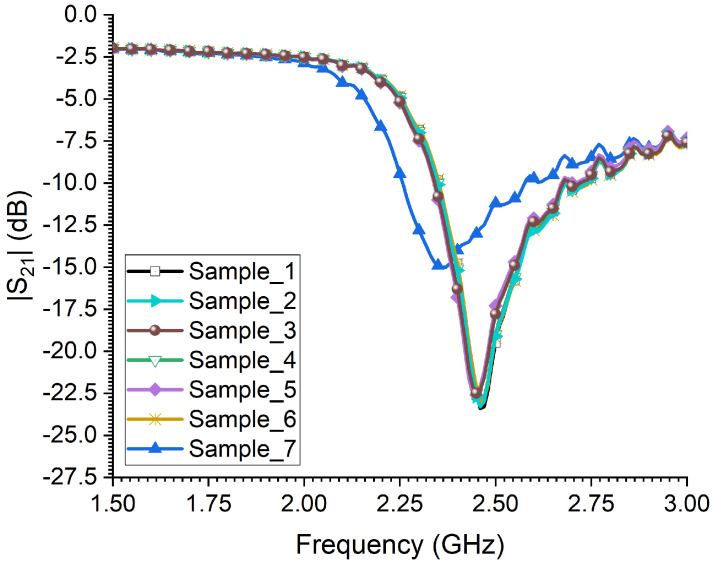
The measured transmission coefficient of the sensor when exposed to 7 larvae RPW insects with different body sizes.

Lastly, the measured transmission coefficient of the CSRR sensor when exposed to seven samples of larvae is presented in Figure 15. Interestingly, not much change in the sensor’s resonance was observed for the larvae samples. This is justified since all samples are of similar body size and qualitatively an indication of similar body losses. Moreover, there is a slightly higher frequency shift of the sensor towards a lower frequency regime for sample 7, which corresponds to a large larva size.

Although the developed microwave CSRR sensor was able to differentiate between the existence of single and dual RPWs within the aperture area of the sensor, we believe that the use of multiple (cascaded) CSRR rings within the sensor could further enhance the possibility of the sensor differentiating more than two RPW pests when placed in the same aperture area of the sensor. Another issue that could limit the high prediction of the current developed CSRR sensor is the prediction of RPW gender due to the random mobility of RPW pests, which could be enhanced with the deployment of artificial-intelligence-based models.

## 4. Conclusions

In this paper, a low-cost microwave CSRR sensor was developed and tested for the identification and monitoring of red palm weevil gender for the first time. The sensor is composed of a two-port transmission line loaded with CSRR inclusion that is etched out from a bottom metallic layer. Full-wave numerical simulations were carried out and demonstrated with experimental results for validation purposes. The microwave sensor is fabricated using standard printed circuit board technology, which makes its deployment as an RPW inspection tool very effective and attractive.

Based on the the simulated results and measured data, the CSRR probe was able to detect variations in RPW gender types, where the the average percentage frequency shift of the sensor for adult RPW samples resulted in being almost 10%, while it was 2.4% when sensing young larvae samples. From the findings of this numerical and experimental study, microwave CSRR-based sensors are found to be promising candidates for the detection and classification of RPW genders due to the reduced cost of fabrication as well as the simplicity of the sensing system setup.

## Figures and Tables

**Table 1:** The dielectric properties of various RPW genders at the 2.45 GHz band estimated from the measured dataset available in [21].

RPW Gender Type	Dielectric Constant	Loss Tangent
Larva	38	12.5
Pupal chamber	39.5	8.5
Adult RPW	7.5	0.125

**Table 2:** Estimated frequency shift of the microwave CSRR sensor when in near-field proximity to various RPW pests in reference to unloaded CSRR sensor.

Sample No.	Male RPW	Female RPW	Larvae
1	3.79	7.03	0.65
2	4.20	7.43	1.33
3	8.24	7.43	1.37
4	11.48	14.30	3.80
5	13.08	19.15	4.61
AVG.	9.46	9.78	2.35

**Table 3:** The CSRR sensor’s resonance frequency when exposed to male, female and larva RPW from front (wings side) and back (belly side).

Surface Type	Male RPW	Female RPW	Larvae
Front	2.32	2.42	2.46
Back	2.26	2.41	2.44
